# α-Conotoxin TxID and [S9K]TxID, α3β4 nAChR Antagonists, Attenuate Expression and Reinstatement of Nicotine-Induced Conditioned Place Preference in Mice

**DOI:** 10.3390/md18120646

**Published:** 2020-12-16

**Authors:** Xiaodan Li, Shen You, Jian Xiong, Yamin Qiao, Jinpeng Yu, Dongting Zhangsun, Sulan Luo

**Affiliations:** 1 Key Laboratory of Tropical Biological Resources of Ministry of Education, Key Laboratory for Marine Drugs of Haikou, School of Life and Pharmaceutical Sciences, Hainan University, Haikou 570228, China; Lixiaodan816@163.com (X.L.); youshen_1992@163.com (S.Y.); xj121968669@163.com (J.X.); qiao_yamin2020@163.com (Y.Q.); zhangsundt@163.com (D.Z.); 2 Medical School, Guangxi University, Nanning 530004, China; mtying0001@126.com

**Keywords:** α3β4 nAChRs, nicotine addiction, α-conotoxin

## Abstract

Tobacco smoking has become a prominent health problem faced around the world. The α3β4 nicotinic acetylcholine receptor (nAChR) is strongly associated with nicotine reward and withdrawal symptom. α-Conotoxin TxID, cloned from Conus textile, is a strong α3β4 nAChR antagonist, which has weak inhibition activity of α6/α3β4 nAChR. Meanwhile, its analogue [S9K]TxID only inhibits α3β4 nAChR (IC_50_ = 6.9 nM), and has no inhibitory activity to other nAChRs. The present experiment investigates the effect of α3β4 nAChR antagonists (TxID and [S9K]TxID) on the expression and reinstatement of nicotine-induced conditioned place preference (CPP) and explores the behaviors of acute nicotine in mice. The animal experimental results showed that TxID and [S9K] TxID could inhibit the expression and reinstatement of CPP, respectively. Moreover, both had no effect in acute nicotine experiment and the locomotor activity in mice. Therefore, these findings reveal that the α3β4 nAChR may be a potential target for anti-nicotine addiction treatment. [S9K]TxID, α3β4 nAChR antagonist, exhibit a superior effect for anti-nicotine addiction, which is promising to develop a novel smoking cessation drug.

## 1. Introduction

Tobacco abuse is an urgent problem in the world and threatens human public health. According to the statistics reported on the global tobacco epidemic 2019, more than 7 million people died from direct smoking, including 1.2 million who were exposed to second-hard smoke every year. [1]. So far, only three pharmacotherapies have been FDA-approved for smoking cessation treatment. The first is α4β2 nAChR partial agonist, varenicline; the other is the atypical antidepressant and monoamine uptake inhibitor, bupropion; and the third is nicotine replacement therapy such as patch, gum, lozenge, inhaler, and nasal spray [2,3,4]. However, the low success rate of the currently available pharmacotherapies to assist in quitting tobacco use clearly highlight the need for more effective treatments for smoking cessation efforts and abstinence.

Nicotine is the main ingredient of tobacco, which is addictive and targets all the nicotinic acetylcholine receptor (nAChR) subtypes without selectivity. Nicotinic acetylcholine receptors (nAChRs) are pentameric ligand-gated ion channels widely distributed in the central and peripheral nervous systems. Nicotine efficiently activates nAChRs presented in brain, resulting in an increase of dopaminergic neuronal excitation in the ventral tegmental area (VTA). As a result, the concentration of dopamine (DA) increases in the nucleus accumbens (NAc), causing people to feel euphoric and satisfied [5]. Human genetic studies and clinical research analyses found that the genetic abnormalities of 15q25 gene cluster increase the risks for tobacco addiction and the early onset of smoking and alcohol abuse. The 15q25 gene cluster can encode the formation of α5, α3, and α4 nAChR [6,7,8]. Previous studies have shown that the α3β4* nAChRs (* indicates the possibility of additional subunits) play an important role in nicotine reward and withdrawal syndrome in mice [9]. AT-1001 bounded to α3β4 nAChRs with high affinity, which inhibits nicotine addiction (self-administration and CPP) in rats and behavioral sensitization in mice [10,11,12,13]. 

α-Conotoxin TxID, discovered from *Conus textile* by gene cloning, is a peptide contains 15 amino acids residues with two disulfide bonds. α-Conotoxin TxID is a strong α3β4 nAChR antagonist (IC_50_ =12.5 nM), which has weak inhibition activity of closely related α6/α3β4 nAChR (IC_50_ = 94 nM) [14]. By using an alanine scanning approach, one mutant [S9A]TxID was found to distinguish these two subtypes, which had a 46-fold discrimination between α3β4 and α6/α3β4 nAChRs [15]. To further improve the selectivity of TxID, the researchers used a series of non-natural amino acids to substitute Serine at position 9 of TxID and found that [S9K]TxID displayed a specific and potent inhibitory effect towards α3β4 nAChRs with an IC_50_ of 6.9 nM [16]. The stabilities of TxID under multiple conditions were evaluated by UPLC based on recommendation of International Conference on Harmonization [17]. The purpose of the present study was to evaluate the effect of α3β4 nAChRs antagonists TxID and [S9K]TxID in nicotine-induced behaviors, by investigating whether TxID and [S9K]TxID would alter the acquisition and relapse of nicotine-induced CPP, and physical acute nicotine behaviors in mice.

## 2. Results

### 2.1. Effect of TxID and [S9K]TxID Alone on Physical Symptoms Caused by Acute Nicotine Exposure

C57BL/6J mice were administrated different doses of TxID or [S9K]TxID alone (i.c.v.) 5 min prior to a single injection (s.c.) of nicotine and evaluated the physical symptoms caused by acute nicotine exposure by hot-plate test and rectal temperature measure (Table 1), After nicotine administration, the hot plate test latency significantly increased (F_6,73_ = 2.499, *p* < 0.05) and the body temperature significantly decreased (F_3,39_ = 13.51, *p* < 0.001). TxID and [S9K]TxID at all doses did not significantly alter the time on hot plate and rectal temperature in mice (*p* > 0.05). 

### 2.2. Effect of TxID and [S9K]TxID on Nicotine Induced CPP Expression 

**Figure 1:**
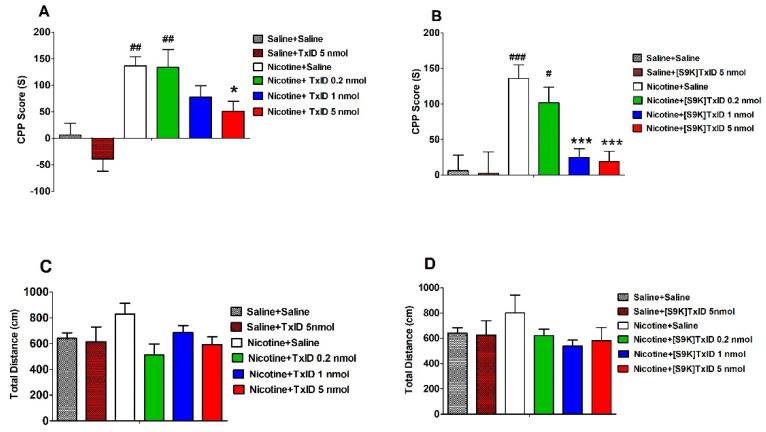
Effect of TxID and [S9K]TxID on nicotine induced CPP expression. (**A**,**B**) are mean (±SEM) CPP score (s), which was the time spent in drug-paired chamber after the injection of Nicotine/TxID/[S9K]TxID minus the initial time spent in drug-paired chamber. (**C**,**D**) are mean (±SEM) total distance (cm) during the 15-min post-conditioning session. Asterisks represent significant difference from the Nicotine + Saline group (* = *p* < 0.05, *** = *p* < 0.001), the pound sign represents significant difference from the Saline + Saline control group (# = *p* < 0.05, ## = *p* < 0.01, ### = *p* < 0.001).

**Figure 2:**
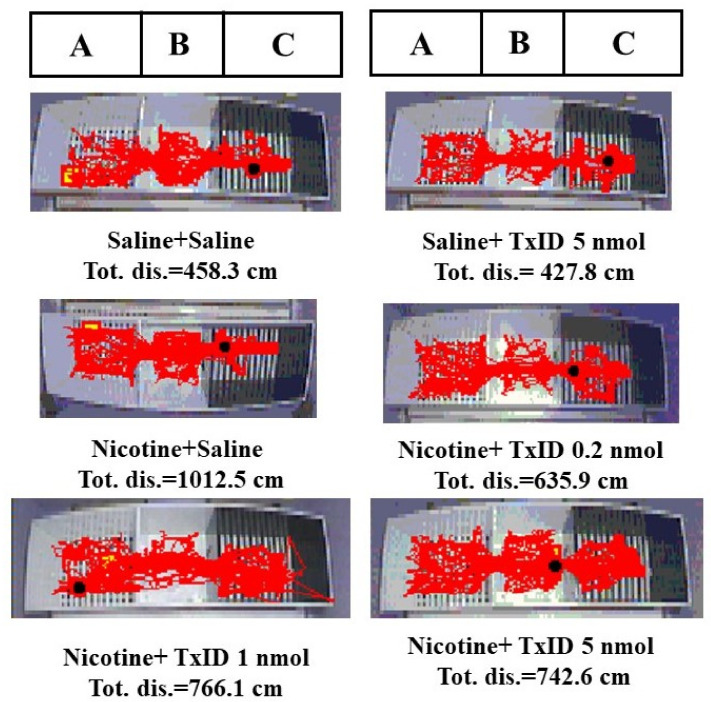
The traces of mice injected with TxID during post-conditioning test. A compartment was white. B compartment was gray and allowed to access. C compartment was black. Nicotine-paired side was white compartment. *n* = 1.

**Figure 3:**
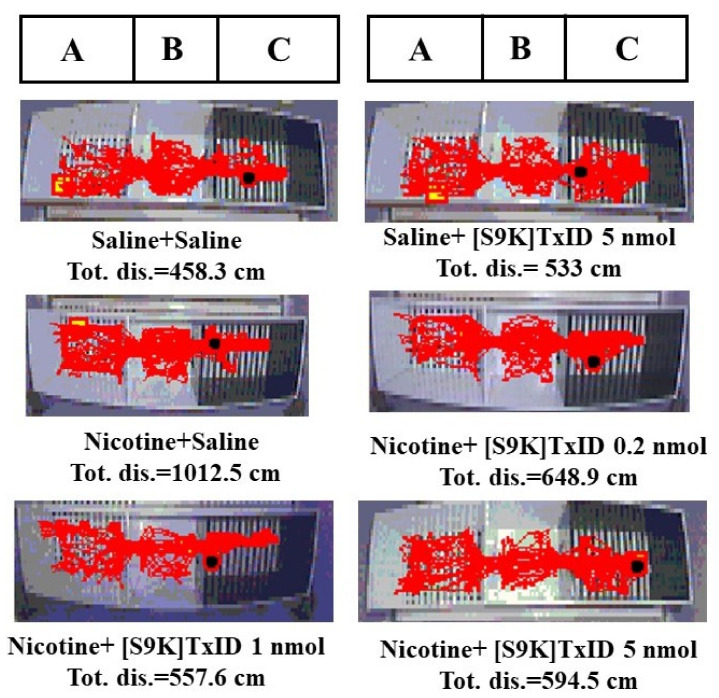
The traces of mice injected with [S9K]TxID during post-conditioning test. A compartment was white. B compartment was gray and allowed to access. C compartment was black. Nicotine-paired side was white compartment. *n* = 1.

After three days of nicotine injection and conditioned training, the time spent in drug-paired compartments of mice injected with nicotine had a significant difference compared to that of the saline treated group (F_8,93_ = 7.198, *p* < 0.001), indicated that the nicotine induced CPP model was successfully established (Table 2). In addition, after surgery the time spent in drug-paired compartments was consistent with post-condition, suggesting that nicotine induced CPP model was robust and stable. The saline induced mice were distributed randomly to the different treatment groups (Saline, TxID 5 nmol and [S9K]TxID 5 nmol). The saline group mice injected with highest dose of TxID and [S9K]TxID had no obvious changes compared with saline group. The nicotine induced mice were distributed randomly to saline and different doses of TxID and [S9K]TxID groups to test the ability to attenuate nicotine induced CPP expression. The α-conotoxin TxID (Figure 1A) and [S9K]TxID (Figure 1B) dose-dependently inhibited the CPP expression. TxID 5 nmol alone could produce a significant effect on blocking the CPP expression relative to Nicotine + Saline (F_5,63_ = 9.194, *p* < 0.05). Similarly, the time spent in the drug-paired compartment of the mice received [S9K]TxID (1 and 5 nmol) significantly decreased compared with mice who received Nicotine + Saline (F_5,57_ = 7.840, *p* < 0.01) demonstrating a significant alleviation of nicotine induced CPP. During post-conditioning test, overall activity was assessed following the injections of TxID (Figure 1C) and [S9K]TxID (Figure 1D). The total distance of 0.5 mg/kg nicotine group increased obviously. A different dose of TxID and [S9K]TxID produced a slight decrease relative to Nicotine + Saline group. However, there was no significant difference among the groups. The tracks of mice movement with white drug-paired chamber are shown in Figure 2 and Figure 3.

### 2.3. Effect of TxID and [S9K]TxID on Nicotine Induced CPP Reinstatement

**Figure 4:**
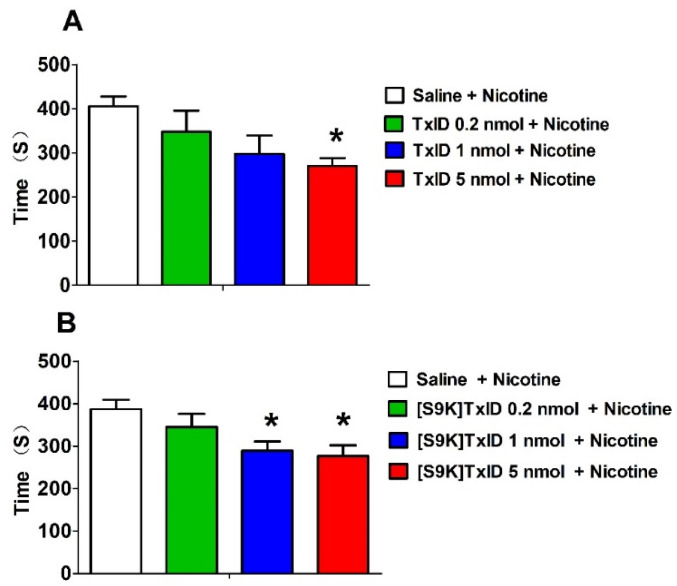
Effects of TxID (**A**) and [S9K]TxID (**B**) on nicotine-induced CPP reinstatement. Data represented mean ± SEM, *n* = 8–10. Time refers to the time spent in drug-paired chamber. Asterisks represent significant difference from the Saline + Nicotine group (* = *p* < 0.05).

Reinstatement is a major problem in smoking cessation. After natural and training extinction, the time spent in the drug-paired chamber decreased to 261.8 ± 4.45 s, which was not more than ± 20% of the base value before CPP (240.6 ± 4.04). The mice showed no obvious preference for drug-paired chamber. Then, the eligible mice were used for reinstatement test, which were injected with TxID or [S9K]TxID 30 min prior to nicotine injection (0.1 mg/kg). The time spent in the drug-paired chamber is shown in Figure 4. The TxID 5 nmol pretreatment could block the CPP reinstatement compared with saline group (F_3,30_ = 3.360, *p* < 0.05). Similarly, the time spent in drug-paired chamber of [S9K]TxID pretreatment with 1 nmol and 5 nmol had a significant decrease compared with saline group (F_3,35_ = 4.555, *p* < 0.05). Although pretreatment with a lower dose did not produce an obvious difference, the effect of decrease tendency appeared. Taken together, TxID and [S9K]TxID dose-dependently inhibited the CPP reinstatement. 

### 2.4. Effect of TxID and [S9K]TxID Alone on Locomotor Activity in Naïve Mice

Spontaneous activity is an index to evaluate the state of excitation or inhibition of the central nervous system. Total distance, central distance/total distance, and central time/total time were used to reflect the activity and exploration characteristics of mice in the new environment [18]. The results of locomotor activity are shown in Table 3. There was no significant difference in each dose of TxID and [S9K]TxID compared with the saline group by One-Way ANOVA. This suggested that TxID or [S9K]TxID may not be involved in the excitation or inhibition of the central nervous system.

## 3. Materials and Methods

### 3.1. Chemical Synthesis of TxID and [S9K]TxID

**Figure 5:**
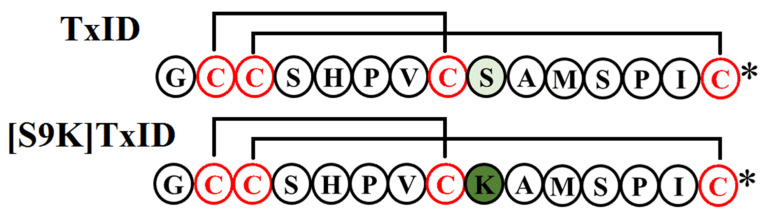
Peptide sequence of TxID and [S9K]TxID. TxID and [S9K]TxID were synthesized with Cys I−III and Cys II−IV connectivities to form the globular isomer. An asterisk (*) indicates a C-terminal amide.

The linear peptides were obtained from GL Biochem (Shanghai, China) using Fmoc chemistry and the cysteine sidechain were protected by acetamidomethyl (Acm) and triphenylmethyl (Trt). The disulfide bonds of the linear peptides were oxidized as previously described [14]. Peptides were kept in 20 mM potassium ferricyanide (K_3_[Fe(CN)_6_]) at room temperature for 45 min to form the first disulfide bond. The closure of the second disulfide bridge was performed by iodine oxidation resulting in formation of TxID and [S9K]TxID (Figure 5). Afterwards, the bicyclic peptide was purified by a reversed-phase Vydac C18 column on the HPLC system and were confirmed by LCMS-IT-TOF mass spectrometer (Shimadzu, Kyoto, Japan).

### 3.2. Animals

Male C57BL/6J mice at the age of 6 weeks, 20–22g) bought from Hunan SJA Laboratory Animal Co., Ltd. (Hunan, China) were used in the present studies. The animals were kept in the SPF animal raising room, Key Laboratory of Tropical Biological Resources, Ministry of Education, University of Hainan. The animal raising rooms were kept a reverse 12 h /12 h light/dark cycle and were maintained at a 23 ± 1 °C humidity-controlled Association. The animals were given food and water ad libitum. Experiments were performed during the light cycle. The International Association for the Study of Pain (IASP) guidelines on the use of awake animals was followed in this study, efforts were also made to minimize the number and discomfort of the animals [19]. This study was approved by the Institutional Animal Care and Use Committee (IACUC).

### 3.3. Intracerebroventricular Surgery

All surgeries were performed using aseptic procedures. C57BL/6J mice were injected with sodium pentobarbital (75 mg/kg i.p.) for anesthesia. Afterwards, a scalp incision was performed in order to expose the bregma. Mice were placed in a stereotaxic device, the unilateral injection sites of cannula implantation were found by using the following coordinates for the lateral ventricle (−0.6 mm AP, +1.3 mm ML, −2 mm DV with respect to bregma). Penicillin was injected after suture. After the mice recovered from the anesthesia, they were housed in separate cages with water and food. After behavioral procedures, brains were harvested to assess cannula placement.

### 3.4. Acute Nicotine Assessment

Sensitivity to thermo stimulation was assessed by the hot plate test (IITC Life Science Co., Ltd., Los Angeles, CA, USA). In brief, mice were placed on the hot plate device, which is a square surrounded by plexiglass which was kept at 55 °C. Mice were observed until they appeared some pain avoidance behaviors such as licking of the paws or jumping. After an acclimatization period, hot plate latency was recorded. To obtain precise baseline intensity, the control mice were tested twice, mice with a control response of 5 to 20 s were selected for the following tests. The mice were injected i.c.v. with TxID and [S9K]TxID or saline 10 min prior to a single injection (s.c.) with 2.5 mg/kg nicotine or saline. Hot plate latency was measured at 5 min following nicotine injection by using the hot-plate tests. To prevent tissue damage, the maximum observation time was set at 40 s.

Rectal temperature was measured with electronic clinical thermometer. Readings were recorded just before and at 5 min after subcutaneous 1.5 mg/kg nicotine injection. The laboratory temperature varied from 23 to 25 °C.

### 3.5. Nicotine-Induced Conditioned Place Preference (CPP)

The CPP apparatus is consisted of a box with three compartments including a black, a white, and a center gray compartment, with an auto-monitoring system obtained from AniLab, Ningbo, China. The white and black compartments (17.38 cm × 13.5 cm × 15 cm each) also had different textures on the floor for the mice to distinguish between the two environments. The left compartment had a white wall with a bar grid floor. The right compartment had a black wall with a mesh floor. The front wall of center compartment was gray, and the floor was neutral (9.8 cm × 13.5 cm × 15 cm). The CPP experiment consisted of the following five different phases. 

#### 3.5.1. Pre-Conditioning

The primary place preference was assessed in the first phase. During this phase, the mice were placed in the central box separately and allowed to access to the entire apparatus for 15 min. The time spent in each of the chambers was recorded at this stage in order to identify the initial preference.

#### 3.5.2. Conditioning

After the pre-conditioning phase, the mice underwent conditioned training, which consisted of 20 min sessions, twice per day (7 h apart) for 3 days. Guillotine doors were closed to confine the mice in one box. The mice were subcutaneously injected twice each day of either nicotine (0.5 mg/kg, morning) or constant dose of normal saline (afternoon) before placed in the white or black boxes and started the timer for 20 min immediately. Meanwhile, the mice in the control group received saline treatment twice per day then were placed in either a black or a white compartment. After each training session, the mice were moved to their home cages.

#### 3.5.3. Post-Conditioning

At 24 h following the last nicotine conditioning trial, the doors were removed so that animals could access these compartments freely for 15 min. The time spent in each compartment was recorded. The group receiving saline injection during the conditioning were used as control group. The robust CPP was indicated by increased the time in the box coupled with the nicotine injection.

#### 3.5.4. Extinction

After post-conditioning, the mice were housed in their home cages for one week, then the mice went through a five-day daily extinction training with no drug injection. The time that mice spent in saline- or nicotine- paired chamber were recorded. Those mice satisfied with the standards were used for the next phase, that the time spent in the nicotine- paired box after extinction was not more than ±20% compared to the pre-conditioning test. The length of time for extinction was approximately two weeks.

#### 3.5.5. Reinstatement

Reinstatement refers to the desire to seek to nicotine again after a prolonged section of nicotine abstinence. When the animals entered to the familiar environment, such as the drug-paired chamber in CPP and re-experience of drug effects, they sought for the drug reinstatement. The mice were administrated with TxID and [S9K]TxID or saline 30 min prior to nicotine (0.1 mg/kg, s.c.) on the reinstatement test day. All the mice were given free access to the whole apparatus for 15 min. The time spent in each chamber was recorded.

### 3.6. Locomotor Activity

Locomotor activity was recorded by the open field apparatus consisted of 4 chambers with an auto-monitoring system (40 × 40 × 35 cm; AniLab, Ningbo, China). The opening area was divided into central area and peripheral area. Before the experiments, the mice were placed into separate activity cages and handled for 5 min to adapt to the apparatus. The mice were given pre-injections of TxID and [S9K]TxID or saline on the test day prior to initiation of the experiment, then were placed in a cage within the locomotor apparatus, and were recorded for 2 h. These behavioral data were analyzed automatically: Locomotor activity, central distance and time (i.e., movement distance and time in the central section of the open field (20 × 20 cm)).

### 3.7. Treatment Groups

α-Conotoxin TxID and [S9K]TxID were dissolved in 0.9% saline to obtain the desired concentration, which were in a volume of 5 μL. For the hot plate, the mice were divided into saline group and treatment groups, which including saline group, TxID or [S9K]TxID (0.2, 1 and 5 nmol) group. Drugs were administered 10 min before injection of nicotine (2.5 mg/kg), latency on hot plate was observed 5 min later. To examine the effects of α-conotoxin TxID and [S9K]TxID on nicotine induced CPP program, the mice were divided into saline group and three treatment groups differing in the dose of TxID or [S9K]TxID (0.2, 1 and 5 nmol) administered 90 min before being placed in the center habituation compartment. For the locomotor activity, the mice were divided into saline group and different treatment groups, which including saline group, TxID and [S9K]TxID (0.2, 1, and 5 nmol) administered being placed in activity cages. Separate groups of animals were used for each experiment.

### 3.8. Statistical Analysis

All data were expressed as mean ± SEM, then the analysis of variance (ANOVA) was used for comparison. The CPP score (s) were considered as the time spent in drug-paired chamber minus the initial time spent in drug-paired chamber. For the acute nicotine induced hot plate, temperature, CPP paradigm, and spontaneous activity, the data were processed using one-way ANOVA and the post hoc Newman–Keuls multiple comparison analyses. The level of significance was set at *p* < 0.05.

## 4. Discussion

The α3β4 nAChR antagonists TxID and [S9K]TxID, significantly and dose-dependently suppressed expression and reinstatement of nicotine induced CPP in mice, but did not induce a place preference or aversion by itself. The minimal effective dose of TxID for reducing the CPP score in the nicotine-paired compartment was 5 nmol per mice, but the reduction in the difference score of [S9K]TxID was more significant. For the acute nicotine exposure, TxID and [S9K]TxID had no obvious effect on hot plate and animal heat under the experimental conditions. Besides, TxID or [S9K]TxID had no effect on the excitation or inhibition of the central nervous system. Previous human genetic [6,20,21] and animal knockout [22,23,24] studies discovered that the α3β4 nAChR displays a vital role in nicotine addiction. Thus, the α3β4 nAChR antagonists are promising for development as smoking cessation medications pharmacotherapy.

Our acute assessment reveal that TxID and [S9K]TxID have no effect in nicotine induced behaviors after acute nicotine injected to mice, even at high dose (5 nmol), which is consistent with previous research on AuIB. Previous studies using β4 nAChR -/- mice suggested that the β4 nAChRs is associated with acute nicotine-induced antinociception and hypothermia [25,26]. The β4 nAChRs can form functional receptors with α3, α6 and α4 nAChR subunits, such as α6β4* or α4β4*, which have different effects. The α3 nAChR expression in the β4 nAChR -/- mice brain were altered, so the compensatory mechanisms to these effects could not be ruled out [22,27]. This experiment shows that the α3β4 nAChRs are not associated with acute nicotine-induced antinociception behaviors in the mice, even at high TxID and [S9K]TxID doses.

These nAChR subtypes, modulating the rewarding properties of nicotine, are differentially expressed. The heterogenous nAChRs α4β2* (* indicates the possibility of additional subunits) and the homologous α7 receptors are the most abundant and widely distributed subtypes, accounting for 95% of total nAChRs in the brain [28,29]. The α6* and α3β4* nAChRs are mostly distributed in certain areas of the hindbrain and midbrain and in the adrenal gland, pineal gland, and most autonomic ganglia [30,31,32]. Many studies have demonstrated the α4β2 nAChRs play an important role in mediating the nicotine enhancement effect [33,34,35]. Researchers evaluated the role of α7 nAChRs in nicotine addiction using α-conotoxin ArlB [VIIL, VI6D] and methyllycaconitine (MLA). The results showed that activation of α7 nAChRs can mediate the nicotine primary reinforcement and prevent smoking relapse triggered [36,37]. Previous studies had shown that the α6β2* nAChRs antagonists could inhibit nicotine addiction in the CPP model and self-administration of the nicotine [38,39,40]. Currently, increasing evidence suggests that the α3β4 nAChRs play an important role in nicotine addiction and withdrawal [9,27,41,42].

In this study, we i.c.v. injected different doses of the α3β4 nAChR antagonists TxID and [S9K]TxID peptide, in nicotine-induced CPP mice. In the nicotine reward assessment, TxID and [S9K]TxID inhibited the expression of nicotine CPP in a dose-dependent manner, suggesting that α3β4* nAChRs plays an important role in nicotine conditioned reward, which is consistent with previous research of AuIB [9]. Although the brain regions involved in the pharmaceutical effects of TxID and [S9K]TxID on nicotine preference were not studied in our experiments, the limited brain distribution of α3β4* nAChRs suggested that the medial habenula-interpeduncular nucleus (MHb-IPN) pathway as a possible site. Previous studies have shown that injection of AuIB or 18-MC (α3β4 nAChRs antagonist) into the IPN decreased intravenous nicotine self-administration [43], and injection of AuIB into the habenular blocked nicotine-induced increases in extracellular dopamine levels in the nucleus accumbens [41]. Other studies strongly supported the role for MHb-IPN α3β4 nAChRs in the aversive components of nicotine addiction [22,44]. In addition, this is the first time to demonstrate that selectively block the α3β4 nAChR (with selective ligands such as TxID and [S9K]TxID) inhibited the nicotine CPP reinstatement. Previous studies showed that α-conotoxin AuIB attenuated the expression of nicotine CPP in C57BL/6 mice, but did not investigate nicotine reinstatement. Gorlich et al. found that α3β4* nAChR activation increased the firing and pacemaking activity of the cholinergic MHb neurons and elevated sensitivity to nicotine in mice during withdrawal [24]. Similarly, AT-1001, α3β4 nAChR partial agonist, reduced yohimbine stress-induced reinstatement of nicotine seeking [45]. Taken together, these studies suggest that antagonism of the α3β4* nAChRs may be a promising approach to control the relapse smoking and help with smoking cessation rates.

The locomotor activity of mice indicated that neither TxID nor [S9K]TxID involve in the excitation or inhibition of the central nervous system. Previous studies have shown that locomotor response to novelty was related to mechanisms of addiction and stress [46,47]. For example, animals with high responses to novelty were found to show higher predisposed to drug self-administration, and a higher sensitivity for natural reinforcers and stressor [48,49]. The highest dose of TxID and [S9K]TxID were injected with saline treated mice which had no obvious changes in CPP test. So the α3β4* nAChRs antagonist, TxID, and [S9K]TxID, could not directly induce drug dependence in mice.

In summary, the highlights of this study are that TxID and [S9K]TxID, the α3β4 nAChR antagonists, blocked the expression and reinstatement of nicotine CPP, and could not produce drug dependence by itself. These results suggest that the α3β4* nAChR are involved in the cholinergic system activation during drug addiction, and that α3β4* nAChRs are potential targets for alleviating important aspects of nicotine dependence. TxID and [S9K]TxID, the potent α3β4 antagonists are promising for development as novel pharmacotherapy for drug abuse and smoking cessation treatment.

## Figures and Tables

**Table 1:** TxID (A) and [S9K]TxID (B) mediated acute nicotine response.

Treatment Group	Dose (nmol)	*n*	Hot Plate (s)	Body Temperature (°C)
Saline-vehicle	-	10	9.31 ± 0.80	36.51 ± 0.23
Vehicle	-	12	20.02 ± 1.57 *	33.35 ± 0.47 ***
TxID	0.2	9	21.11 ± 5.64	——
TxID	1	10	20.65 ± 2.58 *	——
TxID	5	12	20.14 ± 3.40 *	32.77 ± 0.33 ***
[S9K]TxID	0.2	13	19.17 ± 2.06 *	——
[S9K]TxID	1	13	19.65 ± 2.06 *	——
[S9K]TxID	5	10	18.69 ± 2.04 *	33.86 ± 0.54 ***

Each point represents the mean ± SEM. Asterisks represent significant difference compared the saline-negative control group (* = *p* < 0.05, *** = *p* < 0.001).

**Table 2:** Time spent in drug-paired chambers of nicotine induced conditioned place preference (CPP) in mice (s) before the administration of antagonists TxID or [S9K]TxID.

Group		Dose (nmol)	*n*	Pre-Condition	Post-Condition	CPP Score
Saline	Saline	-	10	246.2 ± 23.6	272.1 ± 51.2	25.8 ± 58.4
	TxID	5	10	244.4 ± 31.0	261.8 ± 44.9	17.4 ± 43.9
	[S9K]TxID	5	10	274.4 ± 35.5	275.2 ± 77.6	0.8 ± 73.2
Nicotine	Saline	-	12	247.4 ± 45.1	391.7 ± 64.9 ***	144.3 ± 49.5 ***
	TxID	0.2	10	262.1 ± 33.8	405.9 ± 65.9 ***	143.8 ± 61.8 ***
	TxID	1	12	246.9 ± 37.96	400.6 ± 92.1 ***	153.7 ± 60.3 ***
	TxID	5	15	254.6 ± 40.9	406.6 ± 72.7 ***	151.9 ± 77.5 ***
	[S9K]TxID	0.2	13	242.2 ± 24.5	375.3 ± 63.6 ***	133.1 ± 52.6 ***
	[S9K]TxID	1	11	237.4 ± 22.2	368.8 ± 76.0 ***	131.4 ± 67.9 ***
	[S9K]TxID	5	11	220.6 ± 29.8	350.6 ± 51.1 ***	130.0 ± 39.3 ***

Data represented mean ± SEM, CPP score (s) = the time spent in drug-paired chamber post-conditioning minus pre-conditioning. Asterisks represent significant difference from the saline group (*** = *p* < 0.001).

**Table 3:** Effect of TxID and [S9K]TxID on locomotor activity in naïve mice.

Group	Dose (nmol)	*n*	Total Distances (cm)	Ratios of Center Distance/Total Distance (%)	Ratios of Center Time/Total Time (%)
Saline		10	6329 ± 870.0	0.32 ± 0.021	0.25 ± 0.086
TxID	0.2	12	5741 ± 706.3	0.30 ± 0.022	0.17 ± 0.051
TxID	1	11	6561 ± 1018	0.33 ± 0.018	0.29 ± 0.094
TxID	5	12	6265 ± 902.5	0.30 ± 0.016	0.10 ± 0.035
[S9K]TxID	0.2	11	7239 ± 543.3	0.29 ± 0.019	0.15 ± 0.040
[S9K]TxID	1	15	6344 ± 668.2	0.25 ± 0.015	0.12 ± 0.041
[S9K]TxID	5	14	6828 ± 781.8	0.27 ± 0.016	0.12 ± 0.026

## Abbreviations

nAChR	Nicotinic acetylcholine receptor
CPP	Conditioned place preference
DA	Dopamine
VTA	Ventral tegmental area
NAc	Nucleus accumbens
